# Determination of cyanidin 3-glucoside in rat brain, liver and kidneys by UPLC/MS-MS and its application to a short-term pharmacokinetic study

**DOI:** 10.1038/srep22815

**Published:** 2016-03-11

**Authors:** Stefano Fornasaro, Lovro Ziberna, Mattia Gasperotti, Federica Tramer, Urška Vrhovšek, Fulvio Mattivi, Sabina Passamonti

**Affiliations:** 1 University of Trieste, Department of Life Sciences, via L. Giorgieri 1, 34127 Trieste, Italy; 2 Fondazione Edmund Mach (FEM), Department of Food Quality and Nutrition, Research and Innovation Centre, via E. Mach 1, 38010 San Michele all’Adige, Italy

## Abstract

Anthocyanins exert neuroprotection in various *in vitro* and *in vivo* experimental models. However, no details regarding their brain-related pharmacokinetics are so far available to support claims about their direct neuronal bioactivity as well as to design proper formulations of anthocyanin-based products. To gather this missing piece of knowledge, we intravenously administered a bolus of 668 nmol cyanidin 3-glucoside (C3G) in anaesthetized Wistar rats and shortly after (15 s to 20 min) we collected blood, brain, liver, kidneys and urine samples. Extracts thereof were analysed for C3G and its expected metabolites using UPLC/MS-MS. The data enabled to calculate a set of pharmacokinetics parameters. The main finding was the distinctive, rapid distribution of C3G in the brain, with an apparently constant plasma/brain ratio in the physiologically relevant plasma concentration range (19–355 nM). This is the first report that accurately determines the distribution pattern of C3G in the brain, paving the way to the rational design of future tests of neuroprotection by C3G in animal models and humans.

Cyanidin-3-O-β-glucoside (C3G) belongs to a class of dietary polyphenols known as anthocyanins, which are water-soluble pigments widely distributed among fruits and vegetables, as well as present in red wine^1^. C3G has recently gained attention for its multifunctional neuroprotective effects, including protection against amyloid-beta-peptide-induced toxicity^2^, antioxidant and cognitive promotion activity^3^,^4^, anti-ischemic, and anti-inflammatory properties^5^,^6^. Indeed, chronic consumption of anthocyanins has been correlated with decreased incidence of cognitive decline and neurodegeneration associated with ageing^7^,^8^.

Anthocyanins are quickly absorbed and occur in animal and human plasma and urine in unchanged (i.e. glycosides) and metabolized forms (glucuronidated, sulphated or methylated derivatives)^9^,^10^,^11^,^12^. However, their concentration in blood plasma after the oral intake is very low (nM range)^13^,^14^,^15^. Nevertheless, C3G and other anthocyanins, as well as their derivatives, are detected in tissues such as stomach, skin, small intestine, liver, kidney, lung and eye shortly after administration^16^,^17^,^18^,^19^,^20^. Intact anthocyanins are also able to reach the brain^21^,^22^,^23^,^24^. Importantly, a chronic anthocyanin-enriched diet led to accumulation of C3G and other anthocyanins in the cerebellum, cortex, hippocampus and striatum region of rats brain^23^. Moreover, they were detected in the brain as quickly as 10 minutes from their introduction into the stomach^24^.

*In vitro* studies suggest that short-term incubation can lead to the activation of signalling pathways related to neuroprotection, as observed for ethanol-induced neuronal apoptosis in hippocampal neurons (20 min C3G incubation)^25^. C3G can be accumulated within the brain endothelial cells^26^, and can also cross a blood-brain barrier cellular model in a time-dependent manner, in 1 hour^27^. Despite these clear effects of C3G on the CNS, there is an on-going debate about the degree to which dietary anthocyanins and their metabolites can enter, and eventually accumulate, into the brain^28^. To enable a better understanding of the putative mechanisms of C3G neuroprotective activity *in vivo*, deeper knowledge about its brain-related pharmacokinetics is required. Thus, we focused on short-term C3G pharmacokinetics (PK) (15 s–20 min), after intravenous administration to circumvent inter-individual variability in gastrointestinal absorption. Under these conditions, we observed the rapid distribution of C3G into the brain. The quantitative analysis of C3G and its associated methylated derivatives in rat organs and biofluids, applied to a PK analysis, enabled us to characterize the complex distribution and metabolism of C3G in the rat organism. Indeed, this piece of knowledge is not available in the literature and is therefore reported here in detail for the first time. Recently, there was the direct evidence for the entrance of the flavonoid molecule into the brain parenchyma^29^, thereby opening the rationale for other flavonoids, including anthocyanins, to be localized in neurons or glia. Noteworthy, our experimental strategy involved the least number of animals, in compliance with the 3R principles on animal experimentation.

## Results and Discussion

**Figure 1:**
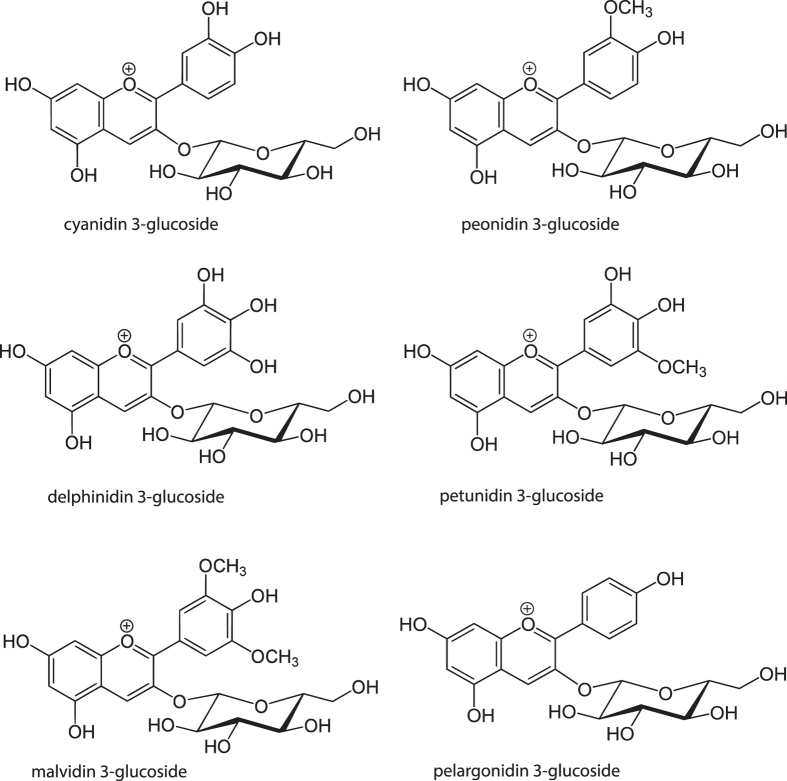
Molecular structures of the analytes.

Table 1 reports the concentrations of C3G and other anthocyanins measured in plasma of anaesthetized rats over a period of 0–20 min after I.V. administration of 668 nmol C3G. The disappearance of C3G was very fast; its biotransformation was rapid and substantial, allowing for the quick appearance of some derivatives in the circulation, such as Peonidin-3-O-β-glucoside (PN3G) and Malvidin-3-O-β-glucoside (M3G). Delphinidin-3-O-β-glucoside (D3G) appeared transiently in plasma only at 2 min. Petunidin-3-O-β-glucoside (PT3G) was detected in plasma only in trace amounts within the first 5 min. These congeners (Fig. 1) may be regarded as the products of hydroxylation and methylation reactions catalysed by cytochrome P450 and catechol O-methyl transferase, respectively^30^. Pelargonidin-3-O-β-glucoside (PG3G) was also found throughout the experiment. Currently, we are unable to speculate about the enzyme activity responsible of this transformation, since no mammalian dehydroxylase is listed in enzyme databases. Recently, we have reported another case of dehydroxylation, i.e. the transformation of urolithin A to urolithin B^28^. No aglycones or conjugated derivatives were detected.

Table 2 reports the concentrations of C3G and other anthocyanins measured in the brain, the liver and the kidneys. C3G distribution and methylation to PN3G were very rapid in these tissues, where PT3G was also found. The amounts recovered in the urine are reported in Table 3. Rat urine showed relatively high amounts of C3G and major metabolites. As much as 15% of the injected dose was recovered 5 min after the injection.

A noteworthy feature is the high rate of C3G disappearance following I.V. administration, thereby making it an analytical challenge to detect plasma C3G at sufficient amounts for its PK parameters to be determined.

### Plasma Pharmacokinetics of C3G

**Figure 2:**
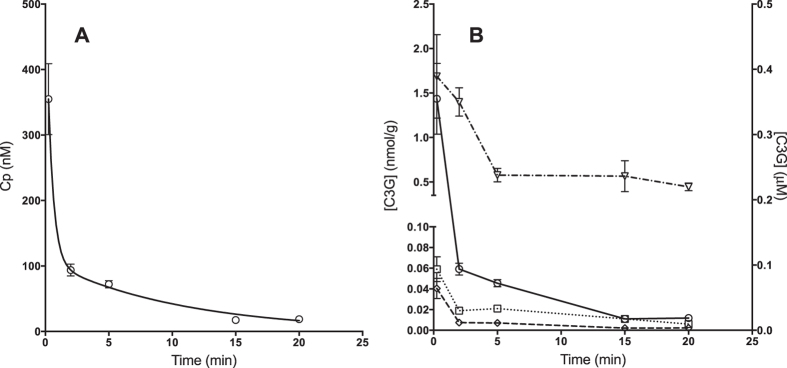
(**A**) Two-compartment model fitting to the C3G plasma concentration-time profile in Wistar rats following intravenous administration of 668 nmol of C3G. ○ Observed; —Predicted. (R^2^ = 0.9992; WSS = 0.14; AIC = −1.93; SC = −3.49). Concentrations are expressed as mean ± SEM (n = 4). (**B**) Concentrations-time courses of C3G in selected tissues: ○ Plasma (μM); ∇ Kidneys, ◻ Liver, ♦ Brain (nmol/g).

The disappearance of C3G from plasma (Fig. 2A) was described by a biphasic curve: a very rapid decline in the first 2 min (distribution phase) that was followed by a slower declining phase (elimination phase). The plasma PK parameters, calculated by both NCA and TCMA, are reported in Table 4. Notably, half-life (t^1^/_2_), volume of distribution at steady state (V_ss_) and total body clearance (CL) were similar if calculated by either method. The extrapolated plasma concentrations of C3G at time zero (C_0_) were calculated by either compartmental or non-compartmental analysis, respectively. The values found were 550 and 429 nM, respectively.

The results allow several novel insights into C3G distribution and metabolism, and they also corroborate findings previously made in the same^31^ or in other rodent species^32^. The mean half-life of C3G was less than 8 min, meaning that ca. 10% of the residual amount of C3G was eliminated per min. Accordingly, we can estimate that the time window needed to accurately describe complete elimination is no more than 1 hour (considering the general rule of a thumb for a complete elimination in 5–6 half-lives, which is in our case 40–50 min).

It is noteworthy that the total body clearance of C3G was 0.49 L/min, a figure that is much higher than the cardiac output (0.10 L/min). Therefore, blood-flow independent elimination can be assumed, e.g. by spontaneous and enzyme-catalysed decomposition to undetectable products, such as the unstable 2,4,6-trihydroxybenzaldehyde^33^, or by direct metabolism occurring in the blood (plasma, red blood cells, and/or endothelial surface) or by a possible lung first-pass effect^19^,^34^.

At the steady state, the volume of distribution of C3G was 4.58 L, which is much greater than total body water^35^, suggesting that C3G is extensively distributed in tissues: when distribution equilibrium is achieved, only 0.24% of C3G is present in plasma, while 99.76% is distributed beyond plasma. As a consequence, the plasma concentration cannot be used as a proxy for predicting the concentration in tissues, with the possible exception of brain, as discussed below.

Other physiological parameters resulting from the two compartment model were: i) the volume in the central compartment (V_1_, 1.21 L); ii) the volume in the peripheral compartment (V_2_, 3.36 L, suggesting that the major fraction of C3G resided in the peripheral compartment); iii) the distribution half-life (t^1^/_2d_, 0.30 min, suggesting that after 18 s, half of the compound had already been cleared from the plasma. In other words, it takes ca. 8 s for distribution to go to 50% completion and between 25 to 42 s for distribution to go to completion); iv) the distribution clearance (CL_d_, 1.76 L/min).

### Peripheral Bioavailability

The mean residence time of C3G in the body (MRT, 9.21 min) is the sum of the mean residence time in both the peripheral space (MRT_P_, 6.78 min) and the central compartment or circulation (MRT_C_, 2.44 min). The peripheral bioavailability (F_AUC_, calculated as MRT_P_/MRT_C_) was 2.77, indicating that C3G molecules tend to spend a longer time (73.51% of its MRT) in the peripheral space than in the central compartment (systemic circulation). This speaks in favour of an accumulation pattern for C3G molecules.

**Figure 3:**
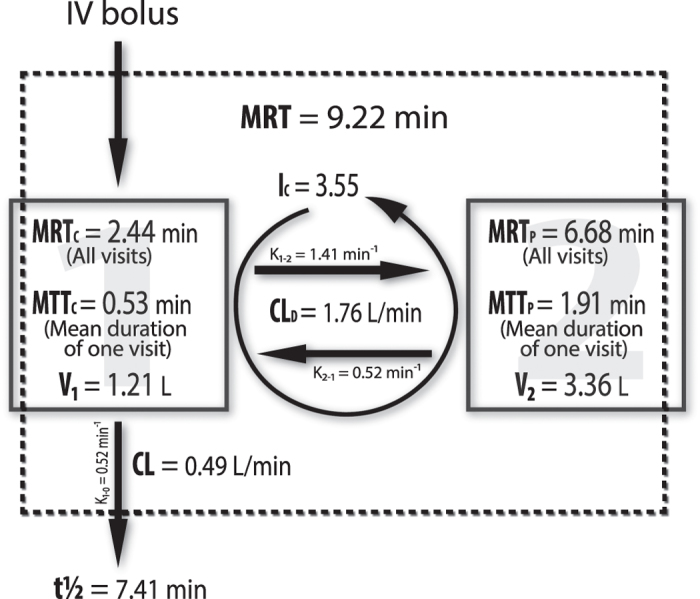
Scheme of a two-compartment, mammillary, open model for C3G disposition after IV administration. See text for details.

The mean transient time of C3G molecules in the peripheral space (MTT_P_, defined as the mean time needed for C3G molecules to return to the central compartment subsequent to entering the peripheral space) was 1.91 min; and the number of circulations made by a molecule on average before being eliminated (I_C_, calculated as MRT_P_/ MTT_P_) was 3.56. In other words, while seven molecules are being distributed from the central to peripheral space, two molecules are being eliminated from the central compartment (Fig. 3).

### Tissue pharmacokinetics of C3G and its metabolites

**Figure 4:**
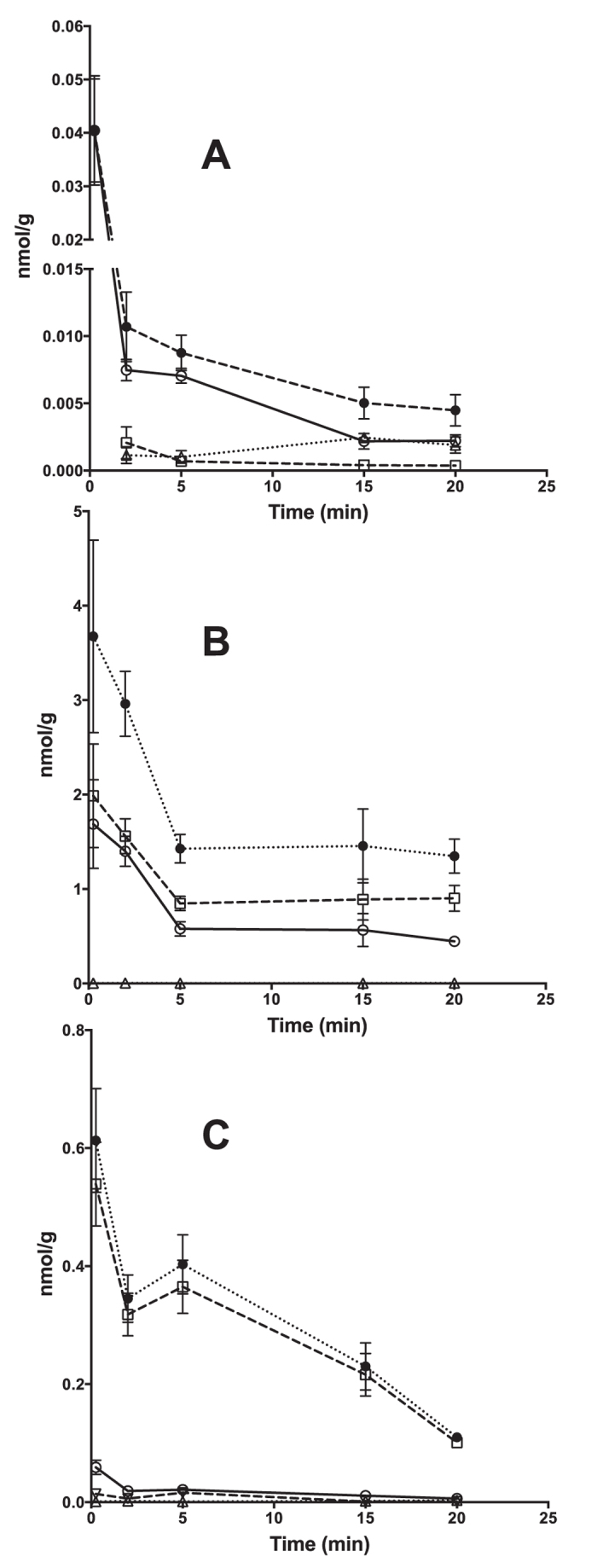
Time-course of C3G and its methylated derivatives in selected tissues. (**A**) Brain; (**B**). Kidneys; (**C**) Liver. ● Total; ○ C3G; ◻ PN3G; Δ PT3G; ∇ isoPN3G.

The concentrations-time courses of C3G in selected tissues are shown in Fig. 2B. C3G, PN3G and PT3G could be detected in brain, liver, and kidneys (Fig. 4 and Table 2). The amounts of C3G found in tissues were in descending order of kidneys (0.44–1.69 nmol/g), liver (5.64–58.61 pmol/g) and brain (2.41–44.11 pmol/g). The AUC values of C3G in the brain and in the liver were in the same concentration range (119.9 and 345.3 pmol/g min, respectively). The AUC value in the kidneys was 14.3 nmol/g min, as reported in Table 2.

Since this experiment was focused on the distributive phase of the concentration-time profile in both the sampled tissues and plasma, the data enabled us to estimate the Mean Transit Time in each specific tissue (MTTi). Under these experimental conditions, this value serves as a good descriptor of tissue distribution differences^36^. Distribution of C3G occurred most quickly in the brain (MTT_b_, 0.43 min), followed by the liver (MTT_l_, 1.43 min) and then the kidneys (MTT_k_, 8.54 min).

The amounts of PN3G recovered in tissues were in descending order of kidneys (0.90–1.99 nmol/g), liver (100.84–539.38 pmol/g) and brain (0.37–2.07 pmol/g). The 4′ positional isomer isoPN3G was detected here as an expected metabolite, based on a critical re-assessment^37^ of previous data. It was here confirmed to be a minor peak, around 2.59% of PN3G, only in the liver. The AUC of PN3G in the kidneys and in the liver was 116.5 times and 32.10 times higher than in plasma, respectively, indicating strong metabolic capacity. For comparison, the AUC in the brain was only 9.78% of that in plasma.

The presence of PN3G in the sampled tissues (MTT_l_, 3.93 min; MTT_k_, 23.11 min; MTT_b_, 8.61 min) was significantly longer than C3G.

### Urinary excretion

Urine concentrations of C3G and methylated derivatives in Wistar rats following intravenous administration of C3G were determined over a period of 0–20 minutes. Urine concentration-time data are listed in Table 3. We detected the urinary excretion after 2 min, which represents the time needed for C3G to undergo uptake, metabolism and excretion in the kidneys. Rat urine showed relatively high amounts of C3G and PN3G. Other anthocyanins were found, though in trace amounts. Moreover, 15% of the injected dose was recovered in 5 min after the injection.

### Significance of C3G in the brain

At 15 sec after the intravenous administration, the C3G levels reached in the brain were 2.41–44.11 pmol/g. Assuming that it is free to diffuse in a water compartment, this value would correspond to approximately 50 nM, a value at which C3G could interact with molecular targets and elicit biological responses. Shortly after, plasma C3G levels rapidly declined, probably due to the rapid disposition of the injected bolus. However, under conditions that simulate the normal peroral consumption, absorption from the gastro-intestinal compartment lasts longer, so it can be speculated that plasma C3G concentration remains elevated for a longer time, as previously shown^9^.

Importantly, the parallel decline of C3G concentrations in the brain and those measured in plasma, as well as the MTT_B_ (0.43 min), also suggests no important retention of the compound in the brain tissue. Such an extensive distribution of C3G, also involving the brain, despite the unfavourable chemical properties of the molecule (hydrophilic molecule) in terms of cell membrane passage, is probably mediated by one or more specific transport mechanisms. Indeed, C3G has been shown to pass the endothelial cell membrane in a short time frame (<1 min)^38^.

As a result, the levels of C3G in the brain linearly correlated with the plasma values, thus providing a measure of the capacity to maintain brain C3G in equilibrium with C3G in the circulation. Furthermore, our data suggest a relatively low inter-individual variability of the blood-brain barrier permeability with respect to C3G under the chosen experimental conditions. Therefore, it can be suggested that the plasma C3G concentrations are a good indicator of the C3G levels in the brain. This implies that further pharmacokinetic studies might be designed accordingly, e.g. by increasing the number of blood samples taken from a single animal, following C3G administration. In the case of oral administration, notably entailing large inter-individual variability of C3G levels in the circulation, any inter-individual variability in plasma levels of C3G should be accompanied by a corresponding variability in C3G brain levels.

**Figure 5:**
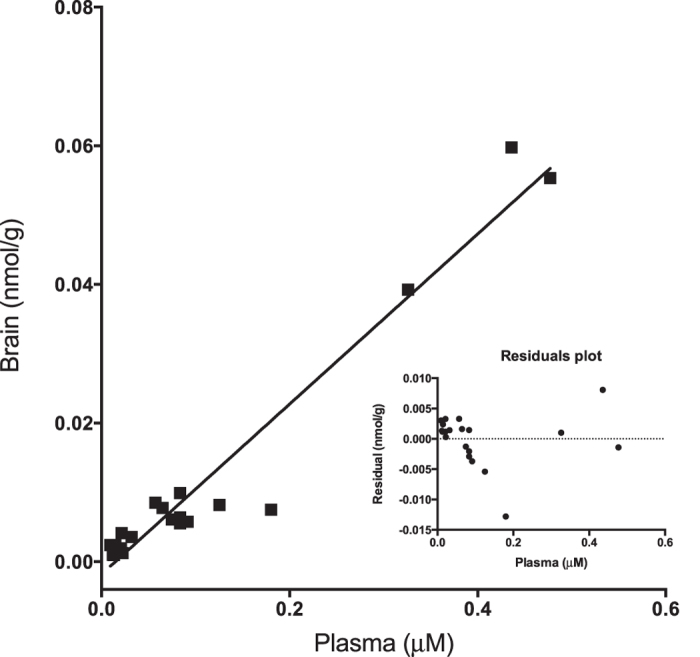
Positive linear correlation (R^2^, 0.9427) between plasma and brain C3G concentrations in individual rats.

To demonstrate the intactness of the blood-brain barrier under the chosen experimental conditions, we showed that the anthocyanin’s profile in the brain (with AUC C3G > PT3G > PN3G, Table 2) was clearly different from that observed in plasma (with AUC C3G > M3G > PN3G > PG3G, Table 1). Indeed, the brain was the only organ in which no substantial metabolism was detected, and a linear correlation between plasma and brain C3G levels was observed (Fig. 5) over a physiologically relevant range of C3G plasma concentrations (18.78–354.84 nM). No correlation could be observed with the other metabolites, once more suggesting the selectivity of the blood-brain barrier.

## Conclusion

Our study provides long-needed quantitative data about C3G distribution in rat biofluids and organs, including the brain. Moreover, the obtained results are now available for designing and interpreting the intervention trials in animals and humans, which are aiming at establishing the link between intake of anthocyanin-rich food or supplements and the protection against age-related cognitive dysfunctions.

## Methods

### Chemicals and reagents

All the chromatographic solvents were HPLC grade or LC-MS grade for the MS experiments. Acetonitrile, acetone, methanol and formic acid were purchased from Sigma Aldrich (Milan, Italy). Isotopically labelled compounds, rosmarinic acid-d7 and cinnamic acid-d5, were purchased from C/D/N Isotopes Inc. (Quebec, Canada). Pure anthocyanins were obtained from Polyphenol Laboratories AS (Sandnes, Norway) and Heparin from Schwarz Pharma (Milan, Italy). All chemicals were used without further purification. Ultra pure Milli-Q water (Merck Millipore, Billerica, MA, USA) was used for the preparation of all solutions. Phosphate buffered saline (PBS) was prepared as following: 6.03 mM Na_2_HPO_4_, 3.91 mM NaH_2_PO_4_ and 139 mM NaCl (Carlo Erba, Milan, Italy) were dissolved in MilliQ water (Millipore) and pH was adjusted to 7.4 with HCl.

### Study design and protocol

The experiment was designed as a one-component pharmacokinetic study with quantitative analysis of the target metabolites in selected organs (liver, kidney and brain) and biofluids (plasma and urine). The principal aim of this study was to characterize the presence of C3G in the anaesthetized rat brain. The three main questions posed were: i) is there any relationship between [C3G] in the blood and in the brain? ii) what is the time needed for C3G to distribute in the brain? iii) for how long is biologically meaningful C3G concentration maintained in the brain?

Twenty-two male Wistar rats (*Rattus Norvegicus*, Harlan Italy S.r.l.), of same age (15 weeks), 293–390 g of body weight, were used. The rats were randomly divided into 5 groups, according to the time that elapsed after intravenous administration of the test compound, and one control group. Each time point was represented by four biological replicates. All animals were allowed to acclimate to the animal facility of the University of Trieste for at least 2 week before studies were initiated. The experimental design was vetted and approved by the bioethical committee of the University of Trieste (internal code 140PAS14), according to the provisions of the European Community Council Directive 2010/63/EU^39^. The methods were carried out in accordance with the approved guidelines. The animals (n = 22) were maintained in cages in a room at 23 ± 2 °C, 50–60% humidity with a 12-hour light-dark cycle. The night before the experiment, food was withdrawn from the cages but water was given *ad libitum*. On the day of the experiment, the rats were anesthetized with intra-peritoneal administration of tiletamine/zolazepam (1:1, 25 mg/kg body weight) and xylazine (10 mg/kg body weight). During anaesthesia (10 min in all cases), the heart and ventilation rate were monitored. The rats were placed on their backs, with the ventral side up and with the legs spread separately on a thermo-isolated support. The penis was extruded by sliding the prepuce downwards. The dorsal penis vein was then seen along both sides of the penis and exactly 10 min after anaesthesia 0.2 mL PBS without (control group) or with (treated group) 668 nmol cyanidin 3-glucoside (C3G) in PBS was injected using a 24-G hypodermic needle. Then the injection site was pressed with a swab for a few seconds, and the glans was encouraged to retract to prevent further bleeding^40^. One min before sacrificing the rats, sodium heparin (0.1 ml, 500 IU) was injected again into the dorsal penis vein, exposed in the same way. Exactly 10 min after anaesthesia and at the corresponding time point after I.V. administration (0.25, 5, 10, 15, 20 min), the rats were sacrificed by decapitation. Blood draining and excision of the organs were carried out according to the literature^20^. In detail, blood was collected from the neck of rats hold upside down, immediately after their decapitation, which happened at the time points sharp. Exsanguination took 5 seconds; laparotomy and excision of the liver and the kidneys took 5 more seconds. The post-decapitation procedures were standardised. Urine was collected through the urinary bladder with a syringe before decapitation. Kidneys, liver and brain were washed with mQ water, immediately frozen in liquid nitrogen and stored at −80 °C.

### Organ collection and extract preparation

Immediately after sampling, 5 mL of blood were transferred into ice-cold (−20 °C), deoxygenated aqueous 95% methanol in a ratio 1:9 (v/v). The urine was weighted and transferred into ice-cold, deoxygenated aqueous 95% methanol at a ratio of 1:9 (w/v). Frozen kidneys, liver and brain were grounded under cryogenic conditions (−196 °C) to 5 μm particles in a CryoMill (Retsch, Germany), using a single 25 mm i. d. steel ball (30 seconds, 25/sec frequency). The pulverized tissue was rapidly transferred (without thawing) into ice-cold, deoxygenated aqueous 95% methanol at a ratio of 1:9 (w/v). Cinnamic acid-d_5_, as internal standard, was dissolved in the aqueous methanol at concentration of 0.1 mg/L for the monitoring of the extraction protocol in biofluids and tissues.

All samples were extracted with an orbital shaker for 10 min at room temperature. The methanol extracts were then centrifuged for 5 min at 3600 rpm at 4 °C, decanted under a stream of nitrogen in 50 mL dark glass vessels and stored at −80 °C. Clean-up of extracted samples in methanol was performed by solid phase extraction (SPE), as previously described^9^. Briefly, 5 mL of extracted samples were evaporated on a rotary evaporator and reconstituted in 10 mL of acidified water. Anthocyanins were extracted by solid-phase adsorption onto a hydrophobic matrix (Sep-Pak C18, 0.35 g, Waters, Milford, MA). Anthocyanins were eluted with methanol, evaporated to dryness and immediately dissolved with 500 μL of methanol:water (50:50). Rosmarinic acid-d7, as internal standard at 1 mg/L, was added in the aqueous methanol for allowing the adjustment of quantitative recovery after sample reconstitution. Samples were filtered through a 0.22 μm PVDF filter (Millipore, Bedford, MA) into HPLC vials for the subsequent quantitative analysis.

### Quantitative analysis

C3G and its derivatives were quantitatively analysed by an ultra performance LC (UPLC) system coupled to a triple quadrupole (TQ) mass spectrometer (see Supplementary information). The UPLC-MS/MS method was chosen to take advantage of the selectivity and sensitivity combined to wide dynamic range of MRM detection in tandem spectrometry, thus allowing the simultaneous quantitation of the main expected metabolites throughout the experiment. A Waters Acquity UPLC (Waters, Manchester, UK) controlled by MassLynx 4.1 was used. Separation of the target metabolites and 2 deuterated internal standards was performed on a reversed phase (RP) ACQUITY UPLC 1.8 μm 2.1 × 100 mm HSS T3 column (Waters) protected with an Acquity UPLC HSS T3 1.8 μm, 2.1 × 5 mm precolumn (Waters), at 40 °C and under a mobile phase flow rate of 0.28 mL/min. Mobile phases of 0.1% formic acid in Milli-Q water (A) and 0.1% formic acid in acetonitrile (B) were used. Chromatographic separation was performed using a multistep linear gradient as follows: 0 min, 5% B; 0–3 min, 5–20% B; 3–4.30 min; 20% B; 4.30–9 min, 20–45% B, 9–11 min, 45–100% B, 11–14 min, 100%; and 14.01–17 min, 5% as equilibration time. Injection volume was 2 μL, and the samples were kept at 4 °C throughout the analysis.

The TQ mass spectrometer used was a Waters Xevo TQ (Milford, Massachusetts, USA) coupled with an electrospray interface. Quantification and confirmation of the anthocyanins were performed using two MRM (multiple reaction monitoring) transition for each compound, using the conditions previously reported^20^. The first transition, corresponding to the most abundant fragment, was used as quantifier ion, and the second as qualifier ion. For calibration, standard anthocyanins were serially diluted in aqueous methanol (50:50), in a concentration range 0.01 μg/L–100 mg/L. The range of calibration curves was obtained on the basis of the linearity of the responses. Acceptable linearity was achieved when the coefficient of calibration curves (R^2^) was at least 0.99. The experimental limit of quantitation (LOQ) in standard solution was at 0.5 ng/mL for C3G, PN3G, M3G and PG3G, and slightly higher for D3G (2 ng/mL) and PT3G (1 ng/mL). Quantitative data were processed with Targetlynx software (Masslynx, Waters). C3G and its derivatives were quantified with the appropriated standard reference with the exception of isoPN3G, expressed as equivalent of PN3G. Details of the UPLC-MS/MS method and quantification are described in^28^.

### Recovery and residual blood correction

According to the published method^20^, the amounts in each matrix were calculated by taking into consideration the appropriate recovery, and by assuming that rat plasma volume is 33.75 μL/g of rat weight^35^. The correction for the residual blood in the brain was performed as proposed in^41^, by subtracting the estimated amount of C3G, PNG and PT3G in the effective plasma space.

### Pharmacokinetics analysis

Pharmacokinetic parameters of I.V. administration of C3G in rats were determined from the mean (n = 4) plasma concentration–time data by both non-compartmental and compartmental analysis as implemented in PK-Solver (version 2.0)^42^. All pharmacokinetic data were expressed as mean ± SEM. The statistical and graphical analyses were accomplished using the software package Prism version 6.0 (GraphPad Software Inc., San Diego, Calif., USA). The statistical significance level was set at p < 0.05.

#### Non-compartmental analysis (NCA)

For NCA, the area under the concentration-time (AUC) curve was calculated using log/linear trapezoidal method from time 0 to the last sampling point 20 min after administration. The PK parameters determined were the concentration at time 0 (C_0_), the terminal elimination rate constant (λ_z_), the terminal elimination half-life (t^1^/_2_), the apparent volume of distribution at terminal phase (V_z_), the relative volume of distribution at steady state (V_ss_), and the total body clearance (CL). The mean time (MT) parameters dealing with the tissue distribution of C3G were calculated from the moments of the concentration-time curve in plasma and specific target tissues, as described^43^. Briefly, Mean residence time (MRT) is the time that a molecule stays in the body, excluding the gastrointestinal tract. MRT was calculated as the ratio of the area under the first moment concentration time curve (AUMC) divided by the area under the zero moment curve (AUC). The terminal elimination half-life (t^1^/_2_) and AUC respective to C3G derivatives were also calculated to determine exposure to any derivatives compared with the parent compound. Maximum plasma concentrations (C_MAX_), and their times of maximal occurrence (T_MAX_) were taken directly from the observed data.

#### Two-Compartment Model Analysis (TCMA)

Mean concentrations of C3G in plasma versus time were further analysed by a two-compartment, mammillary open, IV bolus model, with first order elimination. Data for model fitting were iteratively reweighted by modulating the reciprocal of the squared predicted concentrations (1/C^2^_pre_). The goodness-of-fit was assessed by visual inspection of the residual plots, parameter estimation precision, correlation (R^2^) between the observed and predicted concentration values, weighted sum of squared residuals (WSS), Akaike’s information criterion (AIC), and Schwarz criteria (SC). The lower the WSS, AIC, and SC, the more appropriate is the selected model (Fig. 2). In addition, the information contained in the plasma concentration versus time profile were used to quantify the rate and the extent of the peripheral bioavailability, in terms of mean times spent in the different compartments (MRT_central_ and MRT_peripheral_), number of visits (Ic) in these compartments and mean duration of one visit (MTTC)^44^.

## Additional Information

**How to cite this article**: Fornasaro, S. *et al.* Determination of cyanidin 3-glucoside in rat brain, liver and kidneys by UPLC/MS-MS and its application to a short-term pharmacokinetic study. *Sci. Rep.* **6**, 22815; doi: 10.1038/srep22815 (2016).

## Supplementary Material

Supplementary Information

## Figures and Tables

**Table 1:** Mean plasma concentrations (nM) of C3G and its derivatives in Wistar rats following intravenous administration of 668 nmol of C3G.

Time (min)	C3G	PN3G	PT3G	D3G	M3G	PG3G	TOT
0.25	354.84 ± 54.19	33.68 ± 5.51	2.20 ± 1.05	nd	9.13 ± 3.46	4.50 ± 0.62	404.34 ± 64.83
2	93.80 ± 9.01	13.05 ± 2.35	4.95 ± 3.16	12.81 ± 10.46	95.83 ± 61.71	1.89 ± 0.17	222.33 ± 86.86
5	72.19 ± 5.47	12.31 ± 1.37	1.71 ± 1.39	nd	16.45 ± 13.08	2.01 ± 0.28	104.67 ± 21.58
15	17.50 ± 2.04	3.73 ± 0.94	nd	nd	3.67 ± 0.14	1.90 ± 0.19	26.80 ± 3.31
20	18.78 ± 4.06	3.40 ± 0.22	nd	nd	8.60 ± 3.88	1.55 ± 0.13	32.33 ± 8.29
AUC_0–20_ (nM min)	1165.23	174.77	nsd	nsd	343.45	40.41	1825.57
Half life (min)	6.90	8.16	nsd	nsd	5.29	44.67	5.36

Results are expressed as mean ± SEM (n = 4).

^nd^ Not detected; ^nsd^ data not sufficient to estimate the terminal slope; AUC_0-20_, Area Under the Curve from 0 to 20 min; t^1^/_2_, Half-life; C3G, cyanidin-3-O-glucoside; D3G, delphinidin-3-O-glucoside; PN3G, peonidin-3-O-glucoside; PT3G, Petunidin-3-O-glucoside; M3G, malvidin-3-O-glucoside; PG3G, Pelargonidin-3-O-glucoside. Limits of quantitation (nM) are the following: C3G (1.11), PN3G(1.08), PT3G(2.09), D3G(4.30), M3G(1.01), PG3G (1.15).

**Table 2:** Mean concentrations of C3G and its derivatives in liver, kidneys and brain of Wistar rats following intravenous administration of 668 nmol of C3G.

	**Time (min)**	**C3G (pmol/g)**	**PN3G (pmol/g)**	**PT3G (pmol/g)**	**isoPN3G (pmol/g)**
**Liver**	0.25	56.61 ± 11.79	539.38 ± 71.33	1.23 ± 0.59#	13.86 ± 4.32
	2	19.22 ± 1.99	317.61 ± 35.86	2.42 ± 0.08	6.19 ± 2.09
	5	20.91 ± 2.61	364.74 ± 44.85	1.09 ± 0.52#	15.85 ± 1.82
	15	11.02 ± 2.24	216.10 ± 36.06	1.60 ± 0.44#	1.39 ± 1.14
	20	5.54 ± 0.33	100.84 ± 6.33	3.06 ± 0.19	0.64 ± 0.25#
	**AUC**_**0-t**_ (pmol/g min)	345.29	5610.12	33.85	145.57
	**Half-life** (min)	8.15	9.99	nsd	3.18
	**MTT**_**L**_ (min)	1.43	3.93	nsd	N/A
				
**Kidneys**	**Time (min)**	**C3G (nmol/g)**	**PN3G (nmol/g)**	**PT3G (pmol/g)**	
	0.25	1.69 ± 0.47	1.99 ± 0.55	2.04 ± 0.59#	
	2	1.40 ± 0.16	1.56 ± 0.18	2.82 ± 0.28	
	5	0.58 ± 0.08	0.85 ± 0.08	2.40 ± 0.13	
	15	0.57 ± 0.17	0.89 ± 0.22	2.29 ± 0.07	
	20	0.44 ± 0.04	0.90 ± 0.14	2.53 ± 0.26	
	**AUC**_**0-t**_ (nmol/g min)	14.32	20.36	48.13*	
	**Half-life** (min)	11.66	20.80	135.08	
	**MTT**_**K**_ (min)	8.54	23.11	187.44	
				
**Brain**	**Time (min)**	**C3G (pmol/g)**	**PN3G (pmol/g)**	**PT3G (pmol/g)**	
	0.25	40.46 ± 9.67	nd	nd	
	2	7.48 ± 0.79	2.07 ± 1.18	1.15 ± 0.62#	
	5	7.05 ± 0.55	0.70 ± 0.30#	1.01 ± 0.46#	
	15	2.18 ± 0.58	0.40 ± 0.38#	2.45 ± 0.22	
	20	2.21 ± 0.44	0.37 ± 0.13#	1.90 ± 0.60#	
	**AUC**_**0-t**_ (pmol/g min)	119.89	17.10	32.42	
	**Half-life** (min)	8.80	15.57	nsd	
	**MTT**_**B**_ (min)	0.43	8.61	nsd	

Concentrations are expressed as mean ± SEM (n = 4).

^*^pmol g^–1^ min^–1^; N/A Not applicable; nd, not detected.

^#^value below LOQ; nsd data not sufficient to estimate the terminal slope; AUC_0-inf_, Area under curve; MTT_B_, Mean Transit Time in the brain; MTT_K_, Mean Transit time in kidneys; MTT_L_, Mean Transit Time in the liver.

**Table 3:** Urine concentrations of C3G and its derivatives in Wistar rats following intravenous administration of 668 nmol of C3G.

Time (min)	C3G (nmol/g)	PN3G (nmol/g)	PT3G (pmol/g)	D3G (nmol/g)	M3G (pmol/g)	PG3G (pmol/g)	TOT (nmol/g)
0.25	0.02 ± 0.01	0.01 ± 0.00	4.58 ± 2.84	0.02 ± 0.01	28.35 ± 17.87	1.57 ± 0.40	0.09 ± 0.05
2	2.69 ± 0.82	1.48 ± 0.41	3.30 ± 1.59	0.03 ± 0.00	9.57 ± 4.52	7.92 ± 2.59	4.23 ± 1.24
5	20.20 ± 2.61	6.54 ± 0.33	nd	0.05 ± 0.01	5.15 ± 1.88	47.09 ± 4.16	26.84 ± 2.96
15	11.95 ± 6.53	5.67 ± 2.24	nd	0.02 ± 0.01	3.17 ± 1.64	33.22 ± 16.09	17.68 ± 8.81
20	5.66 ± 1.31	5.16 ± 1.11	nd	0.03 ± 0.01	nd	20.98 ± 5.61	10.87 ± 2.44

nd, Not detected.

**Table 4:** Pharmacokinetic parameters of intravenous administration of 668 nmol of C3G in Wistar rats.

Non-compartmental analysis		Two-compartment model analysis			
Parameter	Value	Parameter	Value	Parameter	Value
λ_z_ (min^−1^)	0.10	a (nM)	442.79	b (nM)	107.68
T_max_ (min)	0.25	α min^–1^	2.29	β min^–1^	0.09
C_max_ (nM)	354.84				
C_0_ (nM)	429.12	k_10_ min^–1^	0.41	Cl_d_ (L/min)	1.76
AUC_0-inf_ (nM*min)	1352.14	k_12_ min^–1^	1.45	CL (L/min)	0.49
AUMC (nM*min^2^)	11789.96	k_21_ min^–1^	0.52	AUC_0-inf_ (nM*min)	1334.49
MRT (min)	8.72	t^1^/_2d_ (min)	0.30	AUMC (nM*min^2^)	12394.67
V_z_ (L)	4.92	t^1^/_2e_ (min)	7.41	MRT (min)	9.22
CL (L/min)	0.49	C_0_ (nM)	550.47	MRT_C_ (min)	2.44
t^1^/_2_ (min)	6.90	V_1_ (L)	1.21	MRT_P_ (min)	6.78
V_ss_ (L)	4.31	V_2_ (L)	3.36	MTT (min)	0.54
		V_ss_ (L)	4.58	MTT_p_ (min)	1.91
		F_AUC_	2.77	I_C_	3.55

a, T_0_ intercept of distribution kinetics; α, Exponent of the polyexponential equation (slope factor); AUC_0-inf_, Area under curve out to infinity; AUMC, Area Under the first Moment Curve from time 0 to infinity; β, Exponent of the polyexponential equation (slope factor); b, T_0_ intercept of elimination phase; C_0_, back-extrapolated drug concentration following rapid bolus iv administration; CL_d_, Distribution clearance; CL, Total body clearance; C_max_, Maximum observed concentration; I_C_, number of circulations; k_10_, rate constant for elimination of drug from central compartment to outside body; k_12_, rate constant for distribution of drug from central compartment to peripheral compartment; k_21_, rate constant for redistribution of drug from peripheral to central compartment; MRT, Mean residence time; MRT_C_, Mean residence time in the central compartment; MRT_P_ , Mean residence time in the peripheral space; MTT, Mean transit time; MTT_p_, Mean Transit Time in the peripheral space; t_max_, Time of occurrence of C_max_; t^1^/_2_, Terminal elimination half-life; t^1^/_2d_, Distribution half-life; t^1^/_2e_, Elimination half-life; V_1_,Volume of central compartment; V_2_, Volume of peripheral compartment; F_AUC_, AUC peripheral bioavailability; V_ss_, Volume of distribution at steady state; λ_z_, First order terminal elimination rate constant.
