# Supplementary material for: Determination of cyanidin 3-glucoside in rat brain, liver and kidneys by UPLC/MS-MS and its application to a short-term pharmacokinetic study
Source: Sci Rep. 2016 Mar 11;6:22815. doi: 10.1038/srep22815 (PMC4786809; doi:10.1038/srep22815)
Supplement: Supplementary Information [file srep22815-s1.pdf]

Determination of cyanidin-3-glucoside in rat brain, liver and kidneys by UPLC/MS-MS and its application to a short-term pharmacokinetic study

Stefano Fornasaro[1], Lovro Ziberna[1], Mattia Gasperotti[2], Federica Tramer[1], Urška Vrhovšek[2], Fulvio Mattivi[2], Sabina Passamonti[1,*]

[1]University of Trieste, Department of Life Sciences, via L. Giorgieri 1, 34127 Trieste, Italy
[2]Fondazione Edmund Mach (FEM), Department of Food Quality and Nutrition, Research and Innovation Centre, via E. Mach 1, 38010 San Michele all'Adige, Italy
*Corresponding author: spassamonti@units.it.

Supplementary information

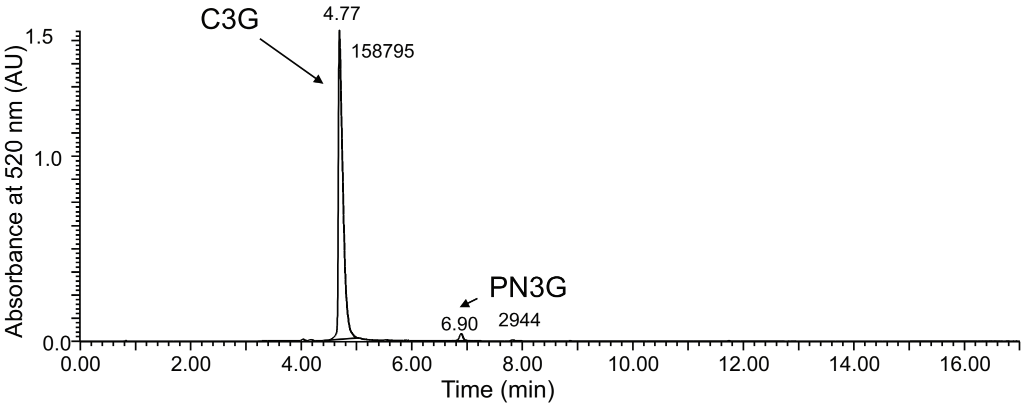

Supplementary Figure S1 Chromatogram of C3G solution (1mg/mL). cyanidin-3-glucoside (C3G) 98,2%; peonidin -3-glucoside (PN3G) 1,8%.

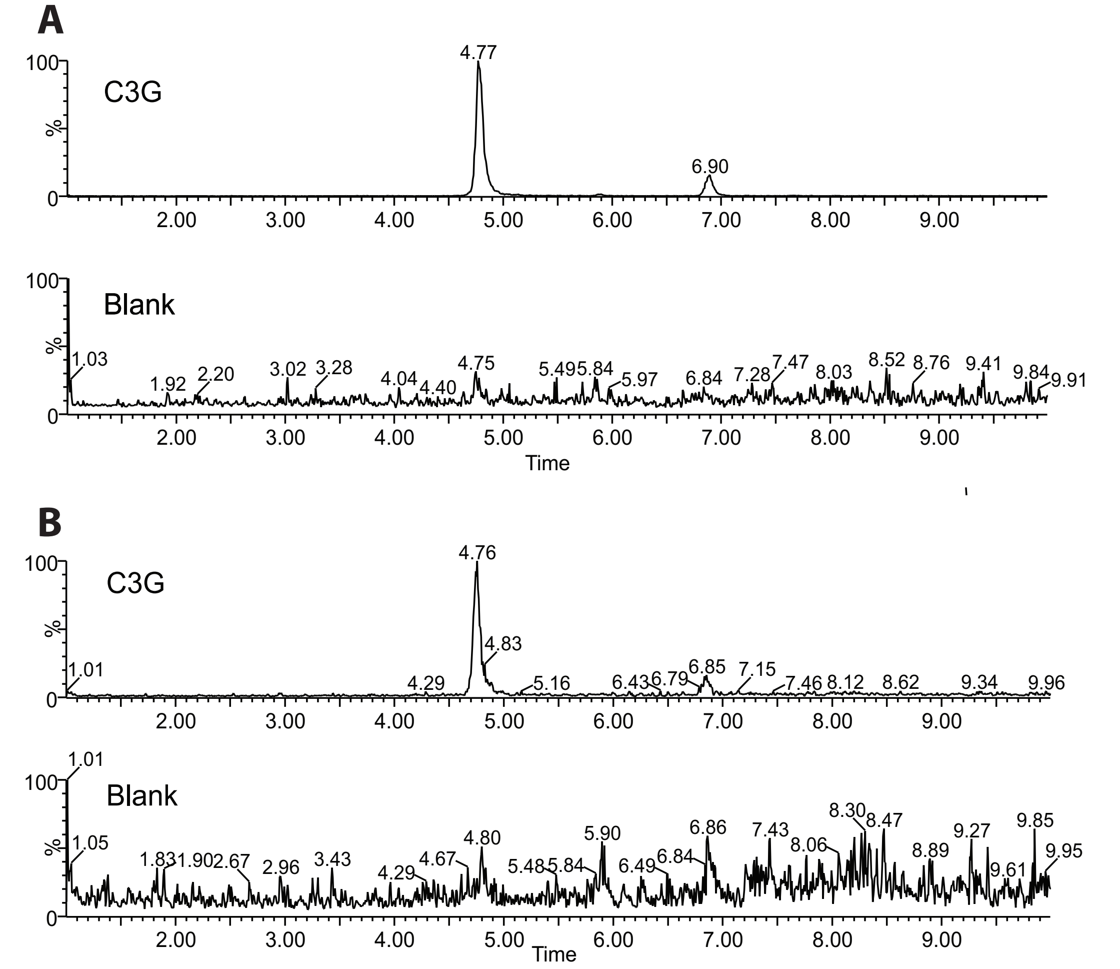

Supplementary Figure S2 Total ion current (TIC) chromatograms for rat blood (A) and brain (B) at the first time point (0,25 min).
